# Supplementary material for: Are carbon emissions trading and green financial instruments synergistic? -Comprehensive quantitative research based on content analysis
Source: PLoS One. 2024 Mar 7;19(3):e0298601. doi: 10.1371/journal.pone.0298601 (PMC10919601; doi:10.1371/journal.pone.0298601)
Supplement: S1 Data — (ZIP) [file pone.0298601.s001.zip › Data/地级市Z_F工作报告.zip!/║■─╧/╔█╤⌠╩╨/└┤╫╘╣1⁄2╓┌║┼í╛┬φ┐╦╩2╛▌═°í┐_2010.docx]

各位代表：现在 ，我代表市人民政府 ，向大会报告工作。请各位代表连同《邵阳市国民经济和社会发展“十二五”规划纲要(草案)》一并审查 ，并请各位政协委员和列席人员提出意见。

一 、“十一五”期间经济社会发展情况

“十一五”时期 ，全市上下按照省委 、省政 府“一化三基”和“四化两型”战略要求 ，克服历史罕见的山洪冰冻灾害和百年不遇的全球金融危机等不利影响 ，坚持科学发展 ，加快后发赶超，推进富民强市 ，全面完成或超额完成了各项目标任务。

五年来 ，经济持续快速增长 ，综合实力显著增强。十一五”时期 ，各项主要指标均提前一年完成目标任务。其中地区生产总值由2005年的340.2亿元增加到2010年的728亿元 ，是“十五”末的2.1倍 ，年均增长12.2%；财政收入由2005年的22.1亿元增加到2010年的51.9亿元 ，是“十五”末的2.3倍 ，年均增长18.2%；全社会固定资产投资由2005年的141.1亿元增加到2010年的610亿元 ，是“十五”末的4.3倍 ，年均增长35.9%；社会消费品零售总额由2005年的118.3亿元增加到2010年的280亿元，是“十五”末的2.4倍 ，年均增长18.8%。

五年来 ，加快转变发展方式 ，经济结构不断优化。新型工业化快速发展。五年中 ，全市规模工业增速和技改投资增速均居全省前列。规模工业增加值由2005年的48.8亿元增加到2010年的205亿元 ，是“十五”末的4.3倍 ，年均增长24.3%。机械 、冶金、食品 、纺织 、医化 、建材 、造纸 、能源等8大优势产业不断壮大 ，规模工业企业由520家增加到872家 ，产值过亿元企业由15家增加到180家 ，其中三一湖汽产值达到35亿元。总投资60亿元的宝庆电厂即将竣工发电 ，总投资13亿元的南方水泥厂点火投产 ，三一湖汽汽车城和湘窖酒业生态城等一批扩改工程抓紧推进。龙山金矿 、江口铁矿和长乐石膏矿等大型矿山开发水平不断提高。新农村建设成效明显。新农村建设办点示范和连片建设扎实推进。农林牧渔增加值由93.1亿元增加到177亿元 ，年均增长6.2%。粮食连年增产丰收 ，总产达到335万吨 ，居全省第一。

养殖业规模居全省首位 ，出栏猪 、牛 、羊 ，分别达到938万头、29万头、40万头 ，总量均居全省第一。农业综合开发成绩显著。农产品加工规模企业由165家增加到277家 ，其中省级农业产业化龙头企业20家 、国家级农业产业化龙头企业1家。全市已建成粮油 、奶业 、生猪 、果蔬 、竹木 、药材和烟草等特色农业生产基地316万亩。优质杂交稻生产 、巩固退耕还林成果 、农村低保和扶贫开发两项制度有效衔接 、高山草山开发等工作走在全国前 列。商贸旅游日益活跃。覆盖城乡的市场服务体系初步形成 ，肯德基 、麦当劳 、步步高和通程电器等大型商贸企业进驻邵阳 ，新办标准化农家店1993家。崀山申遗圆满成功，崀山成为全国第八处和我省第二处世界自然遗产。隆回荣获全国最佳旅游资源城市奖。全市旅游收入达到168.7亿元 ，是“十五”时期的2.4倍。

生态建设初见成效。投入资金30.8亿元 ，完成减排工程项目263个 ，化学需氧量 、二氧化硫和砷等主要污染物削减率分别为11.6%、12.9%和17.4% ，单位国内生产总值能耗累计降低20%。

建成污水处理厂11座 ，城镇污水处理率达到60%以上 ，城镇生活垃圾无害化处理率达到50%以上。植树造林 、公益林保护 、退耕还林和石漠化治理等生态工程进展良好 ，全市森林覆盖率达到56.4% ，西部六县成功创建全国生态示范县。三次产业比由2005年的27.4：30.1：42.5调整为2010年的24.2：38.2：37.6 ，其中二产业比重提高8.1个百分点。

五年来 ，狠抓重大项目建设 ，基础设施明显改善。一批管长远 、打基础和利民生的重大项目相继建成或开工建设。交通建设快速推进。全市交通总投入2416亿元 ，是“十五”期间的6.7倍，超过建国以来历年投资总和。十一五”期间共有8条高速公路开工建设 ，其中邵怀 、邵永和邵衡3条高速建成通车。全市高速公路通车里程达到286.5公里 ，在建的5条高速公路总里程达到223.8公里 ，正式构 成“十”字形骨架。建成和在建国省干线公路项目320个、840.5公里。修建通乡通畅等农村公路1.4万公里 ，全市100%乡镇通油路或水泥路，85%的行政村通油路或水泥路。娄邵铁路扩能改造和沪昆高铁开工建设 ，怀邵衡铁路开工在即 ，铁路即将由单线贯穿变成区域性交通枢纽。各县市1小时上高速和以市区为中心的两小时经济圈已经形成。明年可望实现县县通高速公路。我市被评为全国农村公路建设先进单位和全国交通枢纽城市。武冈和邵东机场列入全国机场建设布局规划。能源建设成绩显著。十一五”期间全市新增水电装机32.7万千瓦。南山风电场开工建设 ，筱溪和晒谷滩等电站蓄水发电。

新建沼气池5.2万口 ，沼气工程达到1369处。我市已经成为全省输变电枢纽城市。城镇面貌日新月异。城市建设步入快速发展轨道 ，实现土地出让价款53.6亿元 ，完成基础设施建设投资150亿元。中心城区建成区面积由42平方公里增加到57平方公里，人口由44万增加到55万 ，全市城镇化率由26.3%提高到31.6%。耕地保护基本国策进一步落实 ，连续九年实现耕地占补平衡。规划引领作用不断加强 ，完成土地利用总体规划修编 ，中心城区建设用地由50平方公里扩大到116平方公里。全市城市总体规划 、县市城镇体系规划修编和村庄布局规划编制工作有序推进 ，完成了历史文化街区 、住房建设 、供水供气和公交等专项规划编制 ，市区控规覆盖率达到100% ，详规覆盖率达到80%。

中心城区实施 了“一带一环”和“九横十纵”新区道路拓展工程，18公里长的邵阳大道全线贯通，21公里长的一环路建成通车；开展 了“一园六路”和资江南岸风光带等提质工程 ，城南 、双清公园和宝庆古城墙 、水府庙戏楼改造工程彰显出城市特色；新建或改建了西湖路 、宝庆路 、魏源东路 、敏州路 、东塔路和资江二桥、西湖桥 、邵水步行桥 、佘湖桥等25条主干道路和4座桥梁；新建了西湖广场 、江北广场 、双龙紫薇园和湘窖文化广场等6个文化休闲广场；完成 了“自来水过江”、天然气置换工程 ，新增供水管网159公里 、供气管网162.8公里。实现房地产开发投资140亿元 ，竣工商品房面积330万平方米；建筑业总产值达到132亿元 ，比2005年翻了一番。各县城和中心城镇建设提速。邵东生态产业园 、新邵酿溪生态园 、武冈铜宝新城和王城公园扩改 、邵阳县夫夷新城综合开发 、隆回路网改造 、洞口大道、崀山大道建设等工程成效明显。两市镇和酿溪镇等15个镇被列为全国或全省重点镇和示范镇 ，廉桥镇和高沙镇等7个镇被列为全省百强镇。

同时 ，城市管理得到加强 ，文明卫生程度不断提升 ，成功创建省级卫生城市。

五年来，改革开放深入推进，发展活力逐步增强。国企改制不断推进，五年完成企业改制154户，国企改制面达到80%，累计盘活企业资产26.9亿元。企业上市工作取得进展，广信造纸和邵阳纺机等6家企业进入省重点上市后备企业资源库。市中小企业信用担保公司成立。城建投体制改革基本完成。矿产资源整顿整合不断加强，第二次全国土地大调查圆满收官。农村综合改革稳步进行，集体林权制度主体改革任务完成。公路管理、文化体制、医药卫生、财税金融、粮食流通、社保医保、投融资体制、事业单位体制、水利管理和资源环境价格等各项改革全面实施。新一轮政府机构改革进展顺利。招商引资连创新高，五年累计引进外资项目133个，利用外资41041万美元，年均增长14.6%；引进内资项目3450个，资金1230亿元，年均增长37.1%。外贸进出口总额实现10.2亿美元，年均增长23.1%。新增境外投资企业123家。

五年来 ，各项事业协调发展 ，人民生活日益殷实。科技创新取得新成绩 ，五年共获得省级以上科技成果20项 ，专利申请量3639件 ，授权量1659件 ，高新技术产值由2005年的27亿元增加到2010年的140亿元 ，我市被列 入“全国知识产权试点城市”。

教育均衡健康发展 ，五年投入资金25.5亿元 ，改造和新建校舍51.4万平方米 ，农村中小学D级危房实现了动态消危。城乡义务教育阶段学杂费全部取消，两基”成果进一步巩固 ，高等教育规模壮大 ，职业教育快速发展 ，民办教育蓬勃兴起 ，素质教育全面推行。公共卫生体系不断健全 ，疾病预防控制体系和突发公共卫生事件医疗救治体系得到加强 ，城市社区卫生服务机构覆盖面达到100%。文化事业繁荣发展 ，大力开展免费送戏送电影下乡活动 ，一批优秀演员和文艺作品获国家级奖励。文化基础设施建设得到加强 ，改建和新建乡镇文化站212个 、农村书屋1447家。绥宁寨市荣膺国家历史文化名镇。湖南祁剧 、邵阳花鼓戏 、洞口棕包脑 、隆回呜哇山歌 、邵阳县蓝印花布和绥宁四八姑娘节列入第二批国家非物质文化遗产保护名录 ，新增国家文物保护单位2处。扫黄打非”不断深入 ，文化市场管理进一步规范。市区数字电视整体转换率达到100% ，村村通”工程扎实推进。全民健身运动蓬勃发展 ，群众性体育特别是中老年体育活动在全省较有影响 ，竞技体育取得新成绩。人口计生工作不断加强 ，人口自然增长率控制在5.6‰以下 ，比“十五”时期下降0.18个千分点。就业和社会保障体系不断完善。五年新增城镇就业岗位24万个 ，城镇登记失业率控制在4.5%以下。全市参加城镇基本养老 、失业 、医疗 、工伤 、生育和新型农村养老保险人数分别达到46.8万人、24.6万人、50.9万人、29.8万人、32.7万人和46.2万人 ，新农合参保率达到94%。五年累计发放各项涉农补贴资金36.3亿元；发放各类社会保障资金134亿元 、社会保障补贴资金5.7亿元 、城镇低保资金2.5亿元 、农村低保资金2.3亿元和移民后扶资金7亿元。建成廉租住房1.4万套、71.3万平方米 ，改造城市和独立工矿棚户区76万平方米 ，发放廉租住房补贴9867万元 ，受益人口2.8万户、6.2万人。改扩建农村敬老院106所。

扶贫开发力度加大。帮助15.3万绝对贫困人口脱贫。解决了100万人的饮水不安全问题。水利建设加强。新建城市防洪堤25.5公里 ，完成368座重点病险水库除险加固。4县84个乡镇建立了山洪灾害预警预报系统。省市两级为民办实事工程全面完成或超额完成。城镇居民人均可支配收入和农民人均纯收入分别达到11686元和3906元 ，年均增长10.9%和9.1%。市场价格基本稳定。全市金融机构存款余额970亿元 ，年均增长18.8% ，其中城乡居民储蓄存款690亿元 ，年均增长16.1%。

五年来，加强民主法制建设 ，社会大局和谐稳定。认真落实市人大及其常委会的决议 ，自觉接受市人大及其常委会的法律监督 、工作监督和市政协的民主监督 ，虚心听取各民主党派、工商联和无党派人士意见 ，密切与工会、共青团和妇联等群众团体的联系。五年共办理市人大代表议案9件 ，建议 、批评和意见1104件 ，市政协提案1713件 ，办复率达到100%。加强基层民主建设 ，推进村民居民自治 ，增强基层民主活力。认真落实国务院《全面依法行政实施纲要》，深入贯彻《行政许可法》 、《政府信息公开条例》和《湖南省行政程序规定》 、《湖南省规范行政裁量权办法》。加快政府职能转变 ，扎实推进政务公开和依法行政 ，全面实施政府绩效评估。五五”普法圆满完成 ，依法治市扎实推进。

廉政建设深入开展 ，发展环境逐步优化。审计力度不断加大 ，财政资金 、民生资金和重大项目资金管理使用日益规范。安全生产形势总体向好 ，重点行业和重点部位监管到位 ，连续四年未发生重大以上安全生产事故。信访工作不断加强 ，人民内部矛盾有效化解。加强社会治安综合治理 ，依法严厉打击各种违法犯罪活动 ，社会大局保持和谐稳定。

国防动员 、民兵预备役和双拥工作成绩显著 ，国家安全和人民防空事业得到加强 ，驻邵解放军和武警部队在抢险救灾 、维护稳定和支持地方建设等方面发挥了重要作用。工商 、物价 、质监、药监 、移民 、市志 、统计 、档案 、贸促 、人事编制 、民族宗教 、外事侨务 、邮政通讯 、气象水文和防震减灾等各项工作都取得了新的进步 ，文联 、残联 、社科联和红十字会等社团组织作用进一步发挥。

各位代表 ，这些成绩的取得 ，是市委统揽全局 、科学决策的结果 ，是市人大 、市政协依法监督 、大力支持的结果 ，是全市各族干部群众锐意进取 、团结奋斗的结果。在此 ，我代表市人民政府，向为邵阳改革发展付出辛勤劳动的全市各族人民 ，向给予政府工作大力支持的人大代表 、政协委员 、各民主党派 、工商联 、无党派人士 、各人民团体 、离退休老同志和社会各界人士 ，向为邵阳发展稳定做出积极贡献的公安干警 、驻邵人民解放军 、武警官兵 ，向所有关心 、支持邵阳发展的中央及省属驻邵单位和来邵投资发展的境内外客商 ，表示衷心的感谢和崇高的敬意！在肯定成绩的同时 ，我们也清醒地看到经济社会生活中还存在诸多不如人意的地方。主要表现为：一是后发赶超压力很大— — — 尽 管“十一五”时期我市各项主要经济指标均呈现前所未有的增速 ，但与全省比较存在较大差距。经济总量在全省占比不高 ，人平均值更是很低。因此 ，在后发赶超过程中 ，不 仅“赶”的难度很大 ，尤 其“超”的负荷更重。二是优化结构任务艰巨— — — 三次产业结构不尽合理 ，农业比重偏高和工业化水平低 、城镇化水平低以及经济外向度低的问题没有根本改变。特别是在工业结构中传统产业升级改造的任务极其繁重。三是财政收支矛盾突出— — — 财政收入虽然五年翻了一番多 ，但各种政策性支出迅速增长 ，保吃饭 、保运转 、保稳定和保发展所需支出不断增长 ，加之重大建设项目和骨干财源企业不多 ，使得财政收支矛盾没有明显缓解。四是发展环境仍然欠佳— — — 基础设施承载能力尚需提高 ，社会稳定压力不可小视 ，政府职能转变和机关作风建设有待加强。总之 ，优化经济发展的软硬环境 ，仍然任重道远。对这些问题 ，我们将采取切实可行措施努力加以解决。

二 、“十二五”的总体思路和奋斗目标

各位代表 ，从今年开始 ，进入“十二五”规划时期。“十二五”是邵阳经济社会发展承前启后和加快赶超的重要时期。虽然我们面临的形势复杂多变 ，但经济向好是总的趋势。首先 ，宏观的发展环境有利。世界经济复苏加快 ，我国国际地位和综合国力显著提升；国家更加重视中部崛起和内陆发展；我省加快推 行“四化两型”战略 ，加大对包括我市在内的大湘西地区的开发扶持力度。其次 ，产业的转移机遇明显。邵阳拥有人力资源和旅游文化生态资源的叠加优势 ，随着交通区位条件的不断改善 ，应当也必将成为承接沿海产业转移和对接产业转型升级的优势区域。再则 ，增长的内生动力较强。这几年基础设施的改善 、优势产业的壮大 、综合实力的提升 ，为加快发展积累了能量。因此 ，我们充分相信：未来五年将是邵阳发展更加精彩和更加辉煌的五年。

“十二五”时期 ，全市经济社会发展总的指导思想是：以科学发展为主题 ，以转变经济发展方式为主线 ，按 照“四化两型”战略部署 ，坚持科学发展 、加快后发赶超 、实现富民强市 ，全面加强优势产业建设 、基础设施建设 、生态文明建设和社会事业建设 ，调整经济结构 ，深化改革开放 ，抓好节能环保 ，保障改善民生 ，确保社会稳定 ，推动经济社会又好又快发展。

— — — 体现经济增长的预期目标再上台阶。主要指标将实现“四个翻 番“”和“四个突破”：即地区生产总值 、规模工业增加值、全社会固定资产投资和社会消费品零售总额实现翻番目标 ，财政总收入突破100亿元 、城镇居民人均可支配收入突破两万元、农民人均纯收入突破6000元和城镇化率突破40%。三次产业比例调整为17.2：46.2：36.6。

— — — 事关长远发展的基础设施更加完善。全市规划建设重点项目515个 ，争取实现投资5000亿元。届时 ，邵阳将完善快速便捷的交通网络 、构建调控有力的水利保障 、发展安全环保的能源供应 、提供统一高效的信息服务。特别是全市交通将形成铁路“三纵三横一连”、高速公 路“三纵三横两连”、民 航“一东一西”和内河水运通畅的全国交通枢纽城市。

— — — 衡定群众利益的幸福指数不断提高。城乡居民人均纯收入年均增长10%以上；价格总水平涨幅控制在适度范围；计划生育工作得到加强；社会就业逐步增加；基本养老 、失业 、医疗 、工伤和生育保险全面覆盖 ，新型农村养老保险逐步铺开。在社会事业发展 、公共服务体系建设和民生改善方面 ，每年兴办一批实事工程和实施一批重点项目 ，让群众直接得到更多的实惠。

— — — 展示自身特色的生态优势日益凸显。森林覆盖率提高到58%以上。单位国内生产总值能耗降低15%。化学需氧量和二氧化硫等主要污染物排放总量累计减少10% ，铅排放量减少10%。城市生活污水集中处理率和生活垃圾无害化处理率均达到100%。中心城区空气质量优良天数达到329天 ，市区饮用水源水质达标率100%。努力建设国家级卫生城市 、森林城市和省级文明城市 、园林城市。

— — — 体现邵阳实力的县域经济竞相崛起。巩固邵东县经济强县优势 ，积极发挥标杆示范效应。大祥 、双清和北塔三区充分利用中心城区有利条件 ，争当加快发展 的“排头兵”。武冈湘桂黔边境中心城市 、新宁国内著名旅游城市建设取得实质性成果。新邵 、邵阳 、隆回 、洞口 、绥宁和城步等县依托优势加快发展 ，形成错位互补和竞相崛起的良好局面。

实现上述奋斗目标，在”十二五“时期我们要突出抓好如下五方面的工作。

1.坚持科学发展，加快后发赶超加速推进新型工业化。突出优势产业发展 ，改造提升传统产业 ，加快由大办工业向办大工业转变的步伐。一是突出产业集群发展。发展壮大机械 、食品和造纸三大主导产业 ，打造主营收入过100亿元集群；改造提升建材 、纺织和化工三大传统产业 ，打造主营收入过50亿元集群；培育扶持医药 、新材料和电子信息三大新兴产业 ，打造主营收入过10亿元集群。紧跟产业发展趋势 ，扶持三一湖汽 、宝庆电厂 、湘窖酒业 、南山乳业 、邵阳纺机 、南方水泥 、立得皮革 、宝兴科肥和本质生物等重点企业做大做强，带动一批中小企业蓬勃发展。二是突出特色园区发展。将江北、邵东 、新邵 、武冈和洞口5家省级经济开发区办成建设规模大和经济效益好的生态科技园区 ，将绥宁 、城步 、新宁 、隆回 、邵阳县和雨溪等6个县级开发区办成产品独特和主业突出的生态特色园区。市本级着重抓好宝庆科技工业园建设。争取全市工业园区总产值占全部工业总产值90%左右。三是突出企业品牌发展。

强化商标注册和品牌培育 ，打造搅拌汽车 、名优曲酒 、纺织机械和营养乳品等具有核心技术和自主品牌的企业。争取到2015年 ，全市核准注册商标总量达到7000件 ，中国驰名商标保有量达到5件 ，湖南省著名商标达到100件。确保20%以上的规模企业通过ISO9000等质量管理体系认证。

大力发展现代农业。一是大力推进农业和农村经济结构调整。提高粮食综合生产能力，全市耕地保持591万亩以上、基本农田保护面积不低于504万亩，稳定粮播面积860万亩、总产330万吨。突出抓好油茶、药材、烟叶、奶业、竹木、水果和延季蔬菜等主导产品生产，形成一县一品或几县一品的农业产业生产带。抓好设施农业科技示范园、蔬菜现代化设施栽培示范县区、全国休闲农业与乡村旅游示范县和示范点建设。继续开发高山草山，促进养殖业健康发展。二是加快发展农产品加工业。重点抓好白酒、乳业、粮油、家禽、林竹、果蔬、豆制品、中药材及生猪和奶(肉)牛等产业链建设。三是继续深化新农村建设。全面完成乡村布局规划编制工作。加强农业农村基础设施建设，重点完善粮食主产区、贫困落后区、库区和移民安置区、退耕还林区等区域基础设施，加快乡村连通道路、教育医疗设施、人畜安全饮水、生态环境保护、农村新能源开发、安全卫生民居和村容村貌整治等工程建设。全市县乡公路全面硬化改造，通村公路硬化率90%以上，基本建成每个乡镇的客运站和村级停靠点。农村自来水受益率提高到60%以上，新建大中型沼气工程50处，沼气入户率达到15%以上。

完成1000个以上村庄整治，建成20个以上省级新农村示范村，打造40个新农村示范片。实施农村劳动力技能培训和农民实用技术培训。引导富余劳动力转移就业。四是建立健全农业服务体系。大力发展农民专业合作社，建好12个大型农机示范基地，提高粮食作物综合机械化水平。建成市本级农产品质检中心和动物疾病预防控制中心，基本建成各县市农产品质检站。加强产地认定和产品认证工作。五是扎实做好农业综合开发。以产粮大县为重点，大力推进农村土地综合整治，集中连片建设高标准农田100万亩，打造粮食生产核心区；以六大主导产业为重点，建设优势产业集约区。与此同时，以加快县域经济为统筹城乡发展着力点，加大政策扶持力度，扩大县级管理权限，增强县域经济活力。

做大做强城镇经济。一是突出规划统筹，构建城镇群体。

按照主体功能规划分区要求，依托湘西地区开发和日益凸显的交通区位优势，构建东部城镇群和西部城镇圈，即以中心城区为中心，以邵阳和隆回县城为辅城，推动新邵等周边县城融城，在东部七县市区构建“1+2+2”聚合型城镇群，尽快打造东部城镇“半小时经济圈”；以武冈市区为中心，洞口、绥宁、城步和新宁县城为支点，在西部五县市构建点轴型城镇圈，尽快打造“一小时经济圈”。扶持经济较发达县市城镇建设，争取邵东、新宁撤县建市和新邵县县改区。全市形成人口规模10至30万人的中小城市7个以上、3万人以上的中心镇20个以上，城镇化率达到40%以上。二是做大中心城区，强化龙头带动。按照“井”字型结构框架，推进“两带两环”建设，即构建资江和邵水风光带，加强沿江(河)基础设施配套，改造小江湖半岛，逐步恢复“宝庆八景”，打造中心城区重要商居带、生态走廊和旅游景观通道；依托沪昆、二广和邵衡高速形成高速外环，把高速外环闭合区内420平方公里区域作为东部城镇群城乡统筹一体化示范区；依托邵坪高速城区连接线、320国道和白马大道形成快速内环，把快速内环闭合区内200平方公里区域作为中心城区建设的主战场。按照交通导向，加快城东工业组团、城南科教组团、城西物流组团和城北工业组团建设，推进城市“东进、南优、北联”；大力推进节地节水节能建设，建成管输天然气入邵项目，实施资江和邵水流域市区段环境综合治理，加快新一轮全市污水和垃圾处理设施建设，实现城镇污水和垃圾处理扩面提标，实施“森林进城、公园下乡”工程，抓好环城森林生态圈和“路边、城边、水边”生态走廊建设。积极创建省级园林城市、文明城市和国家森林城市、卫生城市。开展城乡环境卫生整洁行动。到“十二五”末，中心城区面积达到70平方公里、人口达到70万。

三是突出人本理念，建设宜居城市。加快建立健全基本公共服务体系，制定城市建设投融资规划，多渠道和全方位拓宽融资渠道。更加注重引导民间资本投入城市建设，把更多的人力、财力和物力投入廉租房、公租房、棚改房和安置房等保障性住房建设。不断完善市政公用基础设施，按照城乡一体化的要求，推进城市公交一体化和市政公用设施服务向农村拓展，着力构建以权利公平、机会公平、规则公平和分配公平为主要内容的社会公平保障体系。抓好征地拆迁管理、违法建筑整治和城乡一体化户籍改革，构建新型社会管理模式。

发展壮大第三产业。一是加速做强旅游产业。抓住崀山成功申遗机遇，加速“两圈一带”建设，将邵阳打造成国内外知名生态文化旅游目的地。其中西部生态旅游圈，突出崀山景区提质改造，重点抓好城步南山、绥宁黄桑、武冈云山和洞口罗(草字头+那)溪等项目开发建设；市区历史文化体验与商务休闲旅游圈，重点抓好蔡锷故居综合景区和宝庆古城文化旅游相关项目开发建设；市区至隆回花瑶民俗风情体验休闲旅游带，重点抓好魏源故居、高州温泉、虎形山风情小镇、白水洞、白云岩和龙山森林公园等项目建设。同时，加强配套设施建设，打造“桂林— — —崀山— — — 南山— — — 黄桑— — — 云山— — — 花瑶— — —张家界— — — 神农架”国际旅游大通道和省市精品旅游线路。力争全市年旅游接待人数突破2000万人次，旅游总收入达到220亿元。二是不断繁荣物流产业。建成一批现代物流设施，壮大一批现代物流企业，逐步建立布局合理、装备先进和运作规范的现代物流服务体系。以市区为中心，在城西茶元头周边建设一个综合服务型物流园区，在宝庆科技工业园建设一个与工业配套的物流园区、一个集农资和农产品物流于一体的现代农业物流园区，在新邵雀塘建设一个循环经济和可再生资源加工物流园区，把市区打造成湖南中西部地区重要物资集散中心。抓好市区宝庆物流中心、市名优特新农产品超级市场、邵东星沙物流园、廉桥药都物流园和武冈商都物流园等一批物流项目建设。推进“万村千乡市场工程”和“家电下乡工程”，加快建设一批有辐射力和集聚能力的现代购物超市、配送中心和连锁企业，完善城乡物流网络。三是稳步发展房地产业。使房地产开发投资总量达到150亿元，年竣工房屋面积100万平方米以上，人均居住面积提高到30平方米以上。同时，培植一批房地产经纪和物业管理骨干企业，推动房地产评估、经纪、咨询、测绘和拍卖等行业有序发展。四是培育壮大金融和新兴服务业。完善银行、证券和保险等综合性金融服务体系，探索设立村镇银行和小额贷款公司。深化农村信用社改革，组建区域性商业银行。提高商业银行存贷比。大力推行经纪、策划、代理代办、投资理财和职业介绍等中介服务，规范发展法律、会计、审计、评估和咨询等服务业，不断培育房产、汽车和农副产品等专业会展，支持电子政务、电子商务、远程教育和远程医疗等网络服务业发展。

2.坚持优化发展，突出基础改善完善快速便捷的交通网络。铁路方面 ，完成娄邵铁路扩能改造 ，建好怀邵衡铁路和沪昆客运专线 ，争取启动洛湛铁路邵永段扩能改造工程和建设长沙至娄底和邵阳的长娄邵城际铁路以及邵阳至冷水江的邵冷铁路 ，贵州凯里至邵阳 、永州和郴州的凯郴铁路 ，常德至邵阳和桂林的常桂铁路 ，使全市境内铁路形 成“十”字枢纽 ，通车里程达到310公里。高速公路方面 ，全面建成洞新、安邵 、娄新 、包茂以及邵坪等高速公路 ，开工建设武冈至靖县 、新宁至永州 、白仓至新宁和安铁高速公路邵阳段 ，争取开工城步西岩至贝子河 、邵阳至金石桥和洞口至溆浦等高速公路 ，使全市高速公路达到1016公里 ，通车里程达到830公里 ，实现各县市半小时上高速。干线公路方面 ，提质改造国省干道431公里 、重要经济干线578公里 ，使国省干线二级以上公路总里程达到80%。

农村公路方面 ，重点解决县乡道的危桥 、断头路 、油返砂 、渡边桥和安全保障设施问题 ，抓好大中型水库公路和国有林场通场公路建设 ，实现宜通畅的村100%通畅。机场方面 ，邵东和武冈机场争取建成一个 ，并做好另一个机场前期工作。航运方面 ，抓紧做好资江和邵水的水运恢复工作。

构建调控有力的水利设施。推进安全饮水工程建设，加强农村特别是库区和移民安置区自来水供应设施建设，解决农村150万人饮水不安全问题。完善城市防洪体系，实施市区防洪治涝工程和各县市城市防洪工程，提高防洪标准。抓好大圳、六都寨、威溪、超美和枫树坑等大中型灌区续建配套以及节水灌溉工程建设，推进田间排灌工程、小型灌区和山塘清淤等小型水利设施建设，完成300座小Ⅱ型以上病险水库除险加固工作。做好犬木塘水库修建、木瓜山水库扩建和市区第二水源项目前期工作。搞好全市首次水利普查。

加强安全环保的能源供应。深入开展矿产资源的勘察 、保护和合理开发 ，发展矿产循环经济。全面完成农网和城网改造 ，扎实推进农村电气化 ，实现城乡同网同价。宝庆电厂一期工程投产发电 ，并力争二期工程上马。加快南山风电 、雪峰山风电 、白马山风电 、大云山风电 、龙山风电以及隆回和邵阳县生物质能发电项目建设 ，形成火电与风电等新能源发电相互支撑的电源供应体系。新建500千伏变电站1座 ，新建和扩建220千伏变电站12座、110千伏变电站57座。鼓励扶持清洁环保能源建设 ，完成市区“西气东输二线”管输天然气工程。发展太阳能光伏发电5000千瓦 ，探索地热能 、生物能和小型核电等新型能源。

提供统一高效的信息服务。加快推进物联网和云计算等新技术的研发运用。建成全市统一的电子政务外网平台，实现行政审批电子化和网络化。建设覆盖农村的信息网络，完成新增室外1X宏基站510个，宽带覆盖全部乡村。基本实现自然村通电话和20户以下自然村通广播电视。

3.坚持创新发展，推进改革开放加快科技创新。逐步提高科技经费占财政支出的比例，完善以企业为主体的技术创新体系。抓好知识产权的保护和利用。加强科技创新平台建设，争取建成5个省级企业技术开发中心和30个市级企业技术开发中心。增强农业科技创新能力，积极组建市农业科学院。科技进步对经济贡献率达到50%以上，高新技术产业增加值占工业增加值比重达到40%以上，农业科技产品增加值占农业增加值达到40%以上。

深化体制改革。加快行政审批制度改革，精减行政审批事项；加快投资体制改革，完善投资核准和备案制度，规范政府投资行为；加快财税体制改革，强化部门预算、国库集中收付和政府采购等制度；加快金融体制改革，推进商业银行改革改组，稳步发展各种所有制的中小金融企业；加快企业改革，基本完成国有企业和事业单位改革；加快农村综合改革，积极探索土地经营权流转办法，发展多种形式的适度规模经营，创新征地拆迁安置方式，健全被征地农民合理补偿机制。推进乡镇机构、农村义务教育、农技推广体系、基本药物制度、集体林权制度和水利管理体制等改革。

提升开放水平。加强对外经济技术合作，不断提高经济外向度。研究和建立央企、外企和省企对接机制，积极参与经济全球化和以长株潭、泛珠三角等发达地区为重点的区域经济合作，主动承接产业转移。鼓励支持我市企业到以东南亚为重点的境外创业发展，以邵商品牌打造和提高邵阳的知名度和美誉度。不断扩大电子、皮具、假发、打火机、小五金、液压件和水轮机组等产品的出口份额，积极发展劳务合作和对外工程承包。

大力招商引资。以开展“引老乡回故乡建家乡”活动为载体，突出招商重点，着力承接产业转移和引进战略投资者。积极引导外资投向交通能源、城市建设等基础设施和旅游、农业开发、高新技术产业等重点产业项目。

激励全民创业。出台创业优惠政策，激发全民创业热情。

落实税费减免、创业培训、小额担保贷款和公益性岗位补贴等优惠政策，鼓励能人大胆创业，倡导全员自主创业，引导在外人士回乡创业，支持科技人员开拓创业，进一步激发我市民营经济蓬勃发展的生机和活力。实施人才优先开发战略，用好用活各类人才，培养和引进企业管理人才、专业技术人才、高技能人才和农村实用人才。

4.坚持和谐发展，办好社会事业坚持优先发展教育。强化政府责任，拓宽筹资渠道，加大教育投入，鼓励社会力量办学，建设一批合格学校和寄宿制学校。改善农村办学条件。积极发展学前教育，每个乡镇至少办好一所公立示范幼儿园。根据城区人口分布，科学规划设立小学和幼儿园。基本普及高中阶段教育，优化整合中等职业教育。2015年，高中阶段毛入学率达90%，中等职业教育招生规模与普通高中招生规模大体相当。巩固提升高等教育，积极发展成人教育。尽快启动医专并入邵阳学院，加快组建“湘”字头地方大学。推进邵阳职业技术学院建设，争创全国示范院校。完成邵阳高级技工学校整体搬迁二期工程。申报创办邵阳幼教师范专科学校。进一步优化教育布局，深化教育管理体制和教学体制改革。推进建设“教育强市”工作。

大力繁荣文化事业。推进公共文化设施建设 ，重点抓好市文化艺术中心 、博物馆 、艺术学校艺术楼和祁剧院改造四大工程，加快县乡 村“两馆一院一站一室”建设 ，力争全面完成乡镇 、街道综合文化站和社区文化活动室建设。大力弘扬特色文化 ，唱响地方特色品牌。加强文物申报 、保护 、建设和利用工作。抓好非物质文化遗产传承开发 ，培养本土传人。优化文化产业布局 ，东部地区着重发展文化娱乐 、传媒和出版印刷产业 ，西部地区着重发展文化旅游产业。扎实推进广播电视村村通 、文化信息资源共享、党报阅报栏进社区 、农村电影放映和农家书屋等惠民文化工程建设。加强文化市场监管。推动文化经营性事业单位转企改制。

鼓励社会力量投资文化事业和文化产业。

提高医疗卫生水平。全面完成市县乡村四级医疗卫生机构基础设施体系建设。市本级抓好中心医院异地扩建和市一医院改建工程。各县市至少建好一所高标准的综合医院。抓好城市社区卫生服务机构、乡镇卫生院和村卫生室建设。完成市县两级妇幼保健院、卫生监督所、救援中心和疾控中心改扩建。完 善公立医院基础设施，推进公立医院管理体制改革。加强地方病防治和重大疫病防控。

积极倡导全民健身。建成邵阳体育中心，改造大祥坪体育馆，基本建成各县市区体育馆，逐步配套社区、乡镇和农村体育基础设施。规范发展体育健身、竞赛表演等体育组织和经营实体。大力开展全民健身运动，努力提高竞技体育水平。争取申办2018年省运会。

做好人口计生工作。深入开展“婚育新风进万家活动”，认真落实各项计生奖励优惠政策，提高群众优生优育水平。加大资金投入，完善服务设施，有效解决出生缺陷、出生人口性别比偏高和流动人口管控等难题，切实稳定低生育水平。充分发挥计生协会作用，推进村(居)民自治工作。

5.坚持人本发展，努力改善民生努力做好社会保障工作。加强就业和社会保障工作，千方百计增加就业岗位。积极发展和规范就业中介服务组织和基层社会平台建设，筹建市人力资源市场，形成劳动者自主择业、市场调节就业和政府促进就业相结合的就业机制，推动城乡劳动力合理有序流动。建立健全社会保障体系，加大社会保障投入，逐步实现基本养老、失业、工伤、医疗和生育保险全覆盖，健全农村养老、失地农民保障、合作医疗和农民最低生活保障制度。加强职业技能培训，争取创办邵阳技师学院。加大收入调节力度，逐步扩大中等收入群体比重，不断提高城乡居民收入水平，认真解决低收入群众住房、医疗和子女就学等困难问题。新建廉租房200万平方米和公租房123万平方米，改造双清路、六岭公园、戴家路片区和国有工矿等棚户区300万平方米。发放廉租房租赁补贴2.6亿元。再筹措资金1亿元，基本解决市区拆迁安置历史遗留问题。健全城乡医疗救助体系和优抚对象救助体系，完善临时救助制度，建好市县两级救助中心。加强五保供养，完善城乡低保动态管理。发展多种形式的家政服务、社区医疗和托幼养老等行业。切实保障妇女儿童合法权益，建设市妇女儿童活动中心。提高残疾人和残疾儿童福利水平，建好市县两级残疾人服务中心。

努力做好扶贫开发工作。大力实施基础、产业、科技、生态和社会扶贫“五大工程”，加快贫困地区小康建设步伐。抓好900个村整村推进工作，争取每个村投入财政扶贫资金50万元以上；抓好农业产业化龙头企业建设，每年争取3亿元的扶贫贴息贷款；抓好生产生活条件改善，争取财政扶贫项目资金1.5亿元；围绕实现每个贫困农户培养一位科技带头人和建成一亩经济果园目标，争取财政扶贫项目资金1500万元；围绕35周岁以下富余劳动力转移培训就业，争取财政扶贫项目资金5000万元。争创全国贫困地区劳动力培训转移示范基地。抓好农村低保与扶贫开发两项制度有效衔接，争取每年有12万贫困农民享受低保与扶贫开发政策扶持。

努力做好生态保护工作。继续实施生态林工程，抓好天然林保护、退耕还林、植树造林和中小河流治理以及水土保持，统筹推进城乡绿化美化。加强自然保护区建设，加大生态风景名胜区、饮用水源和生态敏感区保护力度。在资江、邵水和巫水流域营造防护林，积极开展河道清淤整治和截污导流等水环境治理项目，建设“绿色水源”；在铁路、高速公路和国省干道以及景区道路沿线建设防护林带，建设“绿色通道”；在石灰岩山区开展退耕还林和生态移民，加快石漠化治理，建设“绿色山区”；在重点矿区积极推进规模化、集约化和清洁化开采，建设“绿色矿山”。加快建立生态补偿机制，开展环境优美乡镇和生态示范村创建活动。

努力做好安全稳定工作。加强社会治安综合治理和社会管理创新 ，推进社会治安防控体系建设 ，严厉打击各类刑事犯罪。

高度重视安全生产。完善社会利益协调和社会纠纷调处机制 ，拓宽社情民意表达渠道 ，抓好信访和矛盾纠纷调处工作 ，增强维稳风险评估和预警预防能力。完善气象 、水文 、地质灾害 、森林防火和动物防疫等预测预警机制 ，提高防灾减灾能力 ，提高保障公共安全和处置突发事件的能力。加强人防工程建设 ，完善通信警报体系和防空保护体系 ，增强城市防护能力。

努力做好民主法制工作。严格执行人大及其常委会的决议决定，自觉接受政协的民主监督，重视听取各民主党派、工商联和无党派人士的意见和建议，支持工会、共青团、妇联、文联和红十字会等人民团体依照章程开展工作。大力发展基层民主，全面实行政务、厂务、村务和校务公开，依法保障人民群众的知情权、参与权、选择权和监督权，认真办理人大代表建议、批评、意见和政协提案。加强民族宗教和对台、侨务工作，在项目和资金安排方面向民族地区倾斜。以依法行政和公正司法为重点，努力建设法治城市。完善公检法司安基础设施，抓好业务用房和基层“三所一庭”建设。加强普法教育，提高全民法律素质。开展公民道德教育，探索建立社会诚信评价体系。扎实推进政府绩效评估，认真办好实事工程。扎实抓好惩治和预防腐败体系建设，加大案件查处力度，治理行业不正之风，努力建设人民满意政府。不断增强全民国防意识，深入开展“双拥”活动，支持人民解放军和武警部队建设，抓好国防动员、民兵预备役和人防事业，进一步落实军人优待抚恤政策，做好军队转业干部和复员退伍军人接收安置工作。加强档案、地方志、人事编制、外事侨务、防震减灾、关心下一代和无线电管理等工作。

三、切实做好”十二五“开局之年工作

今年是“十二五”时期的开局之年。切实做好今年的各项工作至关重要。

今年全市经济社会发展的主要预期目标是：地区生产总值增长12%；规模工业增加值增长22%；全社会固定资产投资增长30%；社会消费品零售总额增长18%；财政总收入增长15%；城镇居民人均可支配收入增长9%；农民人均纯收入增长8%；引进外资增长10%；引进内资增长15%；进出口总额增长20%；居民消费价格涨幅控制在4%以内；城镇登记失业率控制在4.5%以内；人口自然增长率控制在7‰以内；万元生产总值能耗下降3% ，主要污染物排放总量控制在省政府下达目标以内。

1.全力以赴扩大投入 ，确保较快增长。重点推 进“三个一百”工程 ，即组织实施重点工程项目100个 ，年度投资150亿元；安排前期工作重大项目100个 ，总投资1500亿元 ，项目开工率50%；包装储备重大项目100个 ，投资300亿元以上。一是加强项目前期工作。超前策划一批带动邵阳经济发展的重大项目 ，完善重大项目库。二是大力推进重点工程建设。重点推进怀邵衡铁路 、娄邵铁路扩改 、沪昆客专和洞新高速公路 、安邵高速公路 、邵塘一级公路以及重点工业企业扩改等100个重点工程建设。三是积极融通各类建设投资。加强衔接沟通 ，搞好协调配合 ，加大国家 、省预算内投资以及国家新增投资和国债资金的争取力度。

2.坚持兴工强市战略，增强经济实力。一要调整优化产业结构。在传统产业改造方面，主要是推动冶金、建材、造纸和化工等产业“两型”化改造，引导机械、纺织、食品和竹木等产业集群发展，壮大骨干财源企业。在战略性新兴产业方面，突出培育先进装备制造、电子信息、生物医药、新材料和新能源等产业集约发展，形成新的增长点。在产业配套扩散方面，充分发挥汽车、液压、食品、造纸、纺织和水轮机等重点企业在产业中的龙头作用和带动效应，促进产业同步升级。二要做大做强骨干企业。扶持三一湖汽搅拌车生产达到1.2万台、产值达到60亿元，湘窖酒业完成产值14亿元，宝庆电厂2台机组发电。重点加快神风动力年产7000吨气缸体毛坯铸造、豫湘工贸150万头冷鲜肉加工、宝兴科肥年产15万吨硝酸铵、万事达集团10万吨纱绽纺织材料、云峰水泥日产4500吨熟料新型干法水泥、立得皮革420万张猪牛皮深加工、一纸板厂9000吨宽幅高密度超压绝缘板、本质生物3000吨光合作用催化剂、邵阳纺机新型纤维材料装备研制、维克液压高压柱塞泵、玉新药业黄姜提取物二次深度开发、湘中制药丙戊酸系列产品扩建、恭兵食品湘中辐照中心、圣昌太阳能光伏玻璃和百锐工具整体搬迁等重点产业项目建设力度，集中培植一批税收过亿元企业。三要引导企业入园发展。加快特色园区培育，提升园区产业层次。四要扎实推进节能降耗。健全完善节能统计、监测和考核三位一体体系，加强基层基础工作，为完成“十二五”节能减排任务打好坚实基础。加快云峰水泥和宝兴科肥循环经济试点工作，在建材、化工和冶炼等高耗能行业启动一批循环经济和资源综合利用项目。落实差别电价制度，逐步淘汰技术落后和改造困难的高耗能企业。

3.千方百计筑牢基础，增加农民收入。扎实推进新农村示范建设--新打造20个“产业强、设施好、民风优”的新农村示范片亮点，启动第三轮新农村市级重点示范村建设。大力发展现代农业生产--确保粮食播种面积。抓 好“菜篮子”工程建设。推广规模化养殖。抓好农产品加工龙头企业扩能提质。提高农业综合生产能力— — — 全面完成44座病险水库除险加固任务。

围绕建设100万亩粮食核心区 ，努力打造一批高标准农田。抓好扶贫开发和农村低保两项制度有效衔接。加大农业综合开发力度 ，力争总投入4.8亿元以上。推广各类农机具10万台套 ，落实农机购置补贴8000万元。抓好农村生态环境建设--积极开展人工造林 ，做好集体林权制度改革扫尾工作。发展沼气用户。同时，要加强农民转移就业培训和农业实用技术培训 ，鼓励发展农村二三产业 ，开辟农民创业增收新领域。

4.围绕扩容提质主题，加快城镇建设。一是做好规划编制工作。加快《邵阳市城市总体规划》修编，高标准编制好东部“1+2+2”城市群、420平方公里城乡统筹示范区以及市区100平方公里控制性详规。二是推进中心城区建设。按照“打通主干道、拓宽出入口、建设环城路”的建设思路，突出“两中心一工程十路四区”建设，加快行政中心、文化艺术中心、人防1206工程和建设南路、西湖南路、新华南路、东互通连接线、财神路、桃源路、雪峰南路、邵石南路、中山路、北塔路、学院路和宝庆工业新城、桃花新城、西苑片区、江北新城建设。中心城区建成区面积增加2平方公里，全市城镇化率提高到34%。全面推进“五城同创”活动，抓好西苑公园建设和六岭公园重建工作。同时，加快与新邵县城和周边城镇融城步伐。三是加强城市综合管理。市容管理方面，主要是完成中心城区一至两条路段广告设置示范街改造，解决三眼井和南门口马路市场问题，规范出租车、摩托车、公交车运营和渣土管理秩序。环境卫生方面，突出抓好城中村、小街小巷、机关院落和城乡结合部等薄弱环节的环境治理，保持城市清洁卫生。

灯化亮化方面，重要街道临街单位安装霓虹灯，所有十层以上高层建筑和标志性建筑装配LED灯，力争启动“两江两岸”亮化工程，打造中心城区亮丽风景。

5.不断激发内生活力，搞活商贸流通。一要形成更具活力的体制机制。加快推进政府机构改革，健全政府绩效评估机制，建设服务型政府。深化投资体制改革，做大做强城建投集团。发挥企业主体作用，继续推进国企改制。探索土地经营权流转制度改革，发展多种形式的适度规模经营。全面推进以乡镇机构和农村义务教育为主要内容的农村综合改革。不断推进税收征管改革，理顺完善四级财政管理体制。加强金融风险监管，开展县域金融生态环境评估。二要全面拓宽招商合作领域。组织或参与“深洽会”、“沪洽会”和“湘商大会”等重大招商活动，重点面向长三角、珠三角和港澳台三大地域，加大承接产业转移力度。全年实际利用外资和内资分别增长10%和15%以上，外贸进出口增长20%以上。三要加快做大旅游产业规模。出台《关于进一步加快发展旅游产业的决定》，完善旅游发展规划。抓住崀山申遗成功的机遇 ，着力打造以崀山、云山 、南山 、黄桑和 溪等为基本景点的生态观光旅游区 ，以隆回花瑶 、洞口宗祠 、武冈美食 、城步苗俗和绥宁四八姑娘节等为基本点的民俗文化旅游区 ，以市内三区 、新邵白水洞 、邵东商务休闲和邵阳县相关景区为基本点的环城休闲旅游区 ，加强景点规范化建设 ，做大旅游产业规模。崀山创建5A景区 ，虎形山花瑶创建4A景区 ，蔡锷故居 、双龙紫薇博览园和莲荷生态公园启动国家A级景区创建工作。加快市区五星级酒店建设步伐 ，提高旅行社星级标准 ，搞好邵阳市游客服务中心建设。四要不断扩大城乡消费需求。加快建设湘西南烟草物流中心 、武冈商都物流园和引进沃尔玛百货等现代物流商贸、连锁零售企业。继续实施家电汽车以旧换新、家电汽车摩托车下乡和万村千乡市场工程。全面推进文化、教育、保健、汽车、信贷和房地产等消费升温，增强消费拉动力。

6.扎实有效改善民生 ，建设和谐社会。一是大力为民兴办实事。认真办好省市两级确定的实事工程。时刻关心困难群众和弱势群体 ，就业 、看病和上学等问题。二是发展教科文卫事业。推进教育强市建设 ，确保城乡各级各类各层次教育均衡发展。重点实施好农村中小学危房改造工程 ，合理整合城区教育资源 ，规范城市幼儿教育管理。继续推进在邵高校重组 ，扶持邵阳职业技术学院发展。全市义务教育率达到98%以上 ，基本普及高中阶段教育。完善公共卫生体系。抓好市中心医院异地扩建开工、120紧急救援中心建设和中医院门诊综合楼项目建设 ，做好修建市传染病医院前期工作。继续抓好基本药物制度改革 ，如期启动新邵 、隆回 、洞口和武冈4县市的基本药物新增试点工作。加快博物馆和文化艺术中心等公益性文化设施建设 ，认真开展送书送戏送电影下乡活动。规范管理新闻出版市场 ，大力繁荣文化艺术创作。推进科技进步 ，引领和支撑经济又好又快发展。重点帮助纺织机械 、液压工程 、甾体激素 、绝缘纸和食品工程等5个工程技术研究中心通过省级认定。继续抓好广播电视村村通工程 、农村公益电影工程和卫星电视管理工作。广泛开展群众体育活动，提高竞技体育水平。三是抓好就业和社会保障。深入开展就业援助和就业服务 ，确保新增城镇就业4.5万人和就业困难人员再就业2.8万人。加大社会保险扩面征缴力度，新增参保对象15.5万人。落实被征地农民保险政策，加强农村五保供养工作，提高城乡低保水平。抓好新农合扩面提质工作，使参合率达到95%以上。加大保障性住房建设力度，新建廉租房20.6万平方米，发放租赁补贴3500万元。抓好农业综合开发。加快扶贫整村推进工程。四是认真落实三项基本国策。严格执行现行计生政策，严肃查处违法生育行为，继续稳定低生育水平。加大非法鉴定胎儿性别和非法终止妊娠行为打击力度，降低出生人口性别比。抓好流动人口服务管理，落实各项计生奖励扶助政策。强化“环保优先和环保为民”理念，全面落实“严控、严治和严查”措施，确保城乡环境质量持续改善。全市饮用水质达标率100%，城市优良空气天数300天以上，节能减排指标达到年内进度要求。坚持耕地占补平衡和先补后占制度，保障科学发展用地需求。五是努力保持安全稳定局面。

全面推广洞口县群众工作经验，深入开展社会治安综合治理和社会管理创新，不断加大重点行业和重点领域的隐患排查治理力度，畅通群众信访诉求渠道，提高应对突发公共事件处置能力，巩固和发展我市社会大局和谐稳定的良好势头。

各位代表，做好政府工作，任务艰巨，责任重大。让我们在中共邵阳市委的正确领导下，为全面实施“十二五”规划和出色完成今年各项目标任务而努力奋斗！
